# Intelligence, Correspondence, &c.

**Published:** 1832-01-01

**Authors:** 


### INTELLIGENCE, CORRESPONDENCE, &c.

### PROFESSOR PATTISON.

We are extremely gratified in learning that our transatlantic brethren have healed the wounds inflicted on Mr. PATTISON by his countrymen, by offering him Professorships in two different Universities in the United States. This circumstance speaks volumes as to the talents of Mr. Pattison; but it speaks more than volumes as to the generosity—the almost CHIVALROUS HONOUR and FEELING of the American Medical Profession! In this world, and sorry are we to say, in this country, an *unfortunate* man, whatever be his merits, too often receives a blow from every friend, in order to sink him lower in the sea of misfortune! May glory, fame, and prosperity for ever attend that country, whose HONESTY is not yet engulphed in that sink of mercenary selfishness and corruption which entombed the honour and power of Asia, Egypt, Greece, and Rome—would that we could except modern Europe! N.B. The London University has acknowledged its injustice, by making Mr. Pattison a pecuniary recompence for his wrongs!!

We have received numerous Letters respecting the Nature and Treatment of Cholera Morbus; but it is impossible, in this number of the Journal, to notice them.

Dr. BOURNE's Letter to Sir Henry Halford we have transmitted to a contemporary journal, as it is too long for insertion. Its contents, however, are important in a therapeutical point of view. His reasons for the exhibition of emetics are excellent; and he will see that the remedy is now recommended by the Board of Health.

Mr. MORGAN, of Woodford, proposes urtication, or friction with nettles, in the state of collapse, with the internal use of ammonia, opium, and aromatics. Friction with the common nettle, he observes, is more exciting in paralysis than any other external application whatever. He has frequently used it.

Dr. UWINS' Appeal in favour of the Widow of the late Mr. THOS. WEBBE, Surgeon, of Cold-bath Square, we are unable to insert. This unfortunate female is 71 years of age, and in great distress. Subscriptions will be received by Dr. Uwins, Bedford Row—Mr. Hunter, Solicitor, King's Road, Bedford Row, and Mr. Morgan, Bedford Row.

Mr. SHRAPNELL's paper (with drawings) on the form and structure of the membrana tympani, is under consideration.

Mr. PRESTON on Ligature of the Carotid, for Epilepsy, is received, and will be properly disposed of.

Several communications from our old and esteemed correspondent, Dr. JAMES KENNEDY, of *Ashby de la Zouche*, are received, and will appear in our next number, should the CHOLERA MORBUS spare us so long.

We have been obliged to tax our subscribers, this quarter, with SIXPENCE additional, for 32 pages of closely printed letter-press, EXTRA, containing information worth five times that sum. This, however, will not be repeated.

*** We direct the attention of our readers to the admirable and minute detail, both of the symptoms and pathology of Cholera at Danzig, by Dr. Hamett. It is true that we have published this document somewhat *surreptitiously*, having no authority to do so, from the author nor from the person by whom it was shown to us. We give our enemies (if we have any) full scope for their animadversions on this account. Our general conduct on past occasions, and our motives on this, enable us to lay open a vulnerable point, without much fear of being *ruined* by the procedure.

